# Clinical outcomes of clostridioides difficile infection in the very elderly

**DOI:** 10.1007/s11739-024-03580-0

**Published:** 2024-04-14

**Authors:** Sameer Kassem, Nizar Hijazi, Nili Stein, Adnan Zaina, Mohammad Ganaim

**Affiliations:** 1 Department of Internal Medicine, Lady Davis Carmel Medical Centre, The Ruth and Bruce Rappaport Medical School, Technion Israel Institute of Technology, Michal 7, 3436212 Haifa, Israel; 2 Department of Community Medicine and Epidemiology, Lady Davis Carmel Medical Center, Haifa, Israel; 3 Institute of Endocrinology and Metabolism, Zvulon Medical Center, Clalit Health Services and Azrieli School of Medicine, Bar-Ilan University, Safed, Israel

**Keywords:** Clostridioides difficile, Geriatric, Intensive care unit, Morbidity, Mortality, Very elderly, Elderly, Readmission

## Abstract

**Background:**

Clostridioides difficile infection (CDI) causes considerable morbidity, mortality, and economic cost. Advanced age, prolonged stay in healthcare facility, and exposure to antibiotics are leading risk factors for CDI. Data on CDI clinical outcomes in the very elderly patients are limited.

**Methods:**

A retrospective cohort study of patients hospitalized between 2016 and 2018 with CDI. We evaluated demographic clinical and laboratory parameters. Major clinical outcomes were evaluated including duration of hospital stay, admission to intensive care unit (ICU), in-hospital mortality, 30 days post-discharge mortality, and readmission/mortality composite outcome. We compared patients aged up to 80 years (elderly) to those of 80 years old or more (very elderly).

**Results:**

Of 196 patients included in the study, 112 (57%) were very elderly with a mean age of 86 versus 67 years in the elderly group. The duration of hospital stays, and intensive care unit admission frequency were significantly reduced in the very elderly (13 vs. 22 days *p* = 0.003 and 1.8% vs. 10.7% *p* = 0.01, respectively). No significant difference was found in the frequencies of in-hospital and in 30 days post-discharge mortality.

**Conclusions:**

In our cohort, the duration of hospital stay seemed to be shorter in the very elderly with no increase of in-hospital and post-discharge mortality. Although admitted less frequently to ICU, the in-hospital survival of the very elderly was not adversely affected compared to the elderly, suggesting that very advanced age per se should not be a major factor to consider in determining the prognosis of a patient with CDI.

## Introduction

Clostridioides difficile infection (CDI) is associated with considerable morbidity, mortality, and economic cost [1]. The incidence of CDI is on the rise during the last decades, posing challenges to healthcare organizations [2]. CDI is the leading infectious complication related to hospitalizations or admissions to long-term care facilities [3, 4]. However, despite the common conception linking it to health care facility encounter, a substantial portion of CDI takes place in the community. In a population-based study by Kahnna et al. up to 41% of CDI cases were reported to be community-acquired [5].

The widespread use of antibiotic therapy has been implicated in the increasing incidence of CDI. Clindamycin, fluoroquinolones, cephalosporins, and penicillins were frequently reported in association with CDI [6]. Antibiotics alter the gut flora and provide an advantageous environment for clostridioides difficile (CD) outgrowth, resistance, and the emergence of toxin-producing strains, as was observed in major outbreaks [7]. Virulent CD strains produce mainly two cytotoxins, toxin A and toxin B. The toxins cause damage to colonic epithelial cells, induce polymorphonuclear cell infiltration and swelling of the gut wall [8]. The disrupted mucosal barrier renders the abdominal cavity susceptible to bacterial translocation and systemic infection [9, 10].

CDI-associated diarrhea can be extensive, bloody, and complicated by substantial electrolyte and acid–base abnormalities. Inflammation promotes transluminal fluid loss and secretory diarrhea. In severe cases, the destructive infectious and inflammatory reaction can lead to pseudomembranous colitis, toxic megacolon, and death [11].

In addition to prolonged hospital stay and antibiotic exposure, other factors were identified to increase the risk for CDI, including advanced age, diabetes, obesity, inflammatory bowel disease, poor nutrition, low albumin levels, poor functional status, liver cirrhosis, nasogastric tube feeding, administration of proton pump inhibitors, and chemotherapy [12–16].

CDI is categorized into three levels of severity according to clinical and laboratory parameters: mild to moderate, severe, and severe-complicated CDI (fulminant CDI) [17]. Mild to moderate disease is characterized by abdominal pain and diarrhea but lacks any criteria of severe disease. Severe CDI is characterized by one of the following: fever with a temperature > 38.5 °C, leucocyte count > 15 × 109/L, and rise in serum creatinine > 50% above the baseline. Additional supporting factors, when available are distension of the large intestine, peri colonic fat stranding or colonic wall thickening at imaging. Severe-complicated CDI (or fulminant CD) is defined by the presence of one of the following factors that needs to be attributed to CDI: hypotension, septic shock, elevated serum lactate, ileus, toxic megacolon, bowel perforation or any fulminant course of disease (i.e. rapid deterioration of the patient).

Aging is associated with impaired immune function and increased susceptibility to infections. *T* cell response to foreign antigens is depressed, and the long-term immune memory is diminished [18]. The adverse effect of aging on the humoral immune system is characterized by impaired production of antibodies in response to immunization in geriatric populations [19, 20]. In animal model of aging, neutrophil dysfunction was characterized by disrupted chemotaxis and phagocytosis even in the context of preserved cell count response to infection [21]. Nevertheless, the authors observed that many very elderly patients with CDI recovered and were discharged despite their advanced age and comorbidities. Few studies evaluated the clinical outcomes of CDI specifically amongst patients aged more than 80 years. In this study we aimed to verify our clinical observation. We examined the demographics, laboratory markers of severity, and the clinical outcomes of CDI in the very elderly patients.

## Materials and methods

We conducted a retrospective cohort study that was approved by the institutional review board of Carmel Medical Centre (CMC). CMC is a 500-bed acute care hospital in Haifa, the largest city in north Israel. CMC is part of the Clalit Health Services that provides comprehensive healthcare services in hospital and community-based settings. As such, we have access to patient records during their hospital stays and after discharge. Patients with CDI within the division of internal medicine (4 departments of internal medicine, geriatric, neurology, nephrology, cardiology) in our hospital were grouped in our department (Internal Medicine A) during the specified timeframe. Electronic search of hospitalization records with ICD-9 coding of CDI between 1.1.2016 and 30.1.2018 was performed. Data were extracted from patient records by the investigator (M.G.) on a case-by-case basis. Our cohort consisted only of patients that met all the inclusion criteria. Prior to data analysis, patients were coded by serial numbers, and all identification details were concealed.

Inclusion criteria were age > 18 years, documentation of CDI in the discharge or death letter, documented diarrhea, and positive EIA (C. Diff Quik Check Complete—Techlab) that is verified by PCR test (Xpertt C. Difficile BT—Cepheid). All records with incomplete or missing data or negative test for CDI toxins were excluded from the study.

Information on patient demographics, clinical, and laboratory markers was obtained including age, gender, chronic diseases, disability status, main diagnosis of admission, Charlson comorbidity index score, CDI severity, white blood cell and counts, serum creatinine, albumin, *c*-reactive protein (CRP) levels, and antibiotic treatment for CDI. Major in-hospital clinical outcomes were evaluated. We examined the duration of hospital stays, admission to intensive care unit (ICU) and in-hospital mortality. We also evaluated clinical outcomes after discharge including: 30 days mortality and readmission with recurrent CDI and the composite of readmission and/or mortality at 30 and 60 after discharge.

Two groups of patients were defined: an elderly group aged up to 80 years (80 years not included) and a very elderly group aged 80 years or more. Descriptive analysis was performed on the whole cohort and on the pre-specified age-groups. Inter-group comparative analysis was undertaken for the demographic, laboratory, and clinical outcome parameters.

Categorical variables were summarized with counts and proportions. Continuous variables were summarized with medians and interquartile ranges (IQR). Differences in demographic and clinical characteristics between the elderly and the very elderly were analyzed using Chi-squared test for the categorical variables and Independent *t* test or Mann–Whitney, as appropriate, for the continuous variables.

Multivariable logistic regression was used to identify the risk factors for in-hospital mortality and for the length of hospital stays amongst patients who survived hospitalization. A cutoff of 10 days was defined for a longer hospital stay. Mortality among patients who have been discharged from hospital was estimated using the Kaplan–Meir curves and compared between the two age groups by log rank test. The distribution of time to readmission was estimated by the cumulative incidence function considering mortality as a competing event. Equality of cumulative incidence functions across the two age groups was assessed by Gray’s test.

Statistical analyses were performed using SAS version 9.4 (SAS Institute Inc., Cary NC) and SPSS version 28 (IBM Corp. Released 2022. IBM SPSS Statistics for Windows, version 28.0, 2022, Armonk, NY). In all analyses, *p* ≤ 0.05 for the two-tailed tests was considered statistically significant.

## Results

Initial electronic search revealed 300 patients hospitalized with a diagnosis of CDI during the pre-specified timeframe. We excluded 104 records due to negative EIA/PCR test for CDI toxins. We also excluded records with lack or inaccurate documentation (documentation of diarrhea, records with previous CDI diagnosis that was not deleted and cases that were suspected of having CDI but were found to have another diagnosis). The mean age of the 196 patients in our cohort was 78.5 years, 126 (64%) were females and 70 (36%) males. Eighty-four patients (43%) aged < 80 years and defined as the group of elderly whereas the group of very elderly consisted of 112 (57%) patients. The mean age in the group of elderly was 67 versus 86 years in the group of very elderly.

A background diagnosis of ischemic heart disease, dementia, and disability were more prevalent in the group of very elderly whereas diabetes was more prevalent in the elderly group. Chronic renal failure seemed to be more prevalent in the elderly, but the difference did not reach statistical significance (*p* = 0.051). The Charlson comorbidity index score was significantly higher among the very elderly (6 vs. 8, *p* < 0.001). There was no significant difference in laboratory markers and in CDI severity and in antibiotic treatment for CDI between the groups. Demographic and baseline clinical and laboratory characteristics are presented in Table 1.

**Table 1:** Patient baseline characteristics and laboratory markers

	< 80 years (elderly)	≥ 80 years (very elderly)	*p* value
	*N* = 84	*N* = 112	
Female *N* (%)	57 (67.9)	69 (61.6)	0.366
Hypertension *N* (%)	66 (78.6)	97 (86.6)	0.137
Diabetes *N* (%)	42 (50.0)	39 (34.8)	0.033
IHD *N* (%)	28 (33.3)	66 (58.9)	< 0.001
HF *N* (%)	25 (29.8)	40 (35.7)	0.381
CRF *N* (%)	44 (52.4)	43 (38.4)	0.051
CVA *N* (%)	22 (26.2)	32 (28.6)	0.712
COPD *N* (%)	13 (15.5)	14 (12.5)	0.55
Dementia *N* (%)	12 (14.3)	42 (37.5)	< 0.001
Disability *N* (%)	31 (36.9)	67 (59.8)	0.001
Malignancy *N* (%)	33 (39.3)	33 (29.5)	0.15
Leucocytes median (IQR)	11 (7.1; 15)	12 (9; 16.9)	0.07
Neutrophils % median (IQR)	81 (73; 88)	83 (73; 88)	0.312
Creatinine median (IQR)	1.3 (0.8; 2.4)	1.2 (0.8; 2)	0.554
Albumin median (IQR)	2.7 (2.3; 3.3)	2.9 (2.4; 3.2)	0.436
CRP median (IQR)	9.1 (2.9; 19.5)	9.9 (5; 15)	0.924
Charlson score median (IQR)	6 (4; 9) *N* = 79	8 (6; 9) *N* = 98	< 0.001
Disease severity			0.282
Mild/moderate	53 (63.1)	59 (52.7)	
Severe	22 (26.2)	41 (36.6)	
Severe complicated	9 (10.7)	12 (10.7)	
CDI treatment			0.272
Vancomycin	25 (30.1)	42 (37.5)	
Metronidazole	46 (55.4)	49 (43.8)	
Vancomycin + Metronidazole	12 (14.5)	21 (18.8)	

*IHD* ischemic heart disease, *HF* heart failure, *CRF* chronic renal failure, *CVA* cerebrovascular accident, *COPD* chronic obstructive pulmonary disease, *CRP* *c*-reactive protein

There was no significant difference in admission due to CDI-related colitis between the elderly and the very elderly groups (*p* = 0.65). Frequent main diagnoses of our cohort are displayed in Table 2.

**Table 2:** Main diagnosis of patients

	< 80 years (elderly)	≥ 80 years (very elderly)
Main diagnosis	*N* = 84 (%)	*N* = 112 (%)
CDI	23 (27)	34 (30)
UTI	6 (7)	24 (21)
Pneumonia	13 (15)	17 (15)
Infection—other	12 (14)	9 (8)
CHF exacerbation	8 (10)	3 (3)
ARF	5 (6)	3 (3)
Other	17 (20)	22 (20)

*CDI* clostridioides difficile infection, *UTI* urinary tract infection, *CHF* congestive heart failure, *ARF* acute renal failure. (“other” = chronic obstructive pulmonary disease, acute coronary syndrome, cerebrovascular accident, anemia, malignancy)

*p* = 0.65

**Fig. 1:**
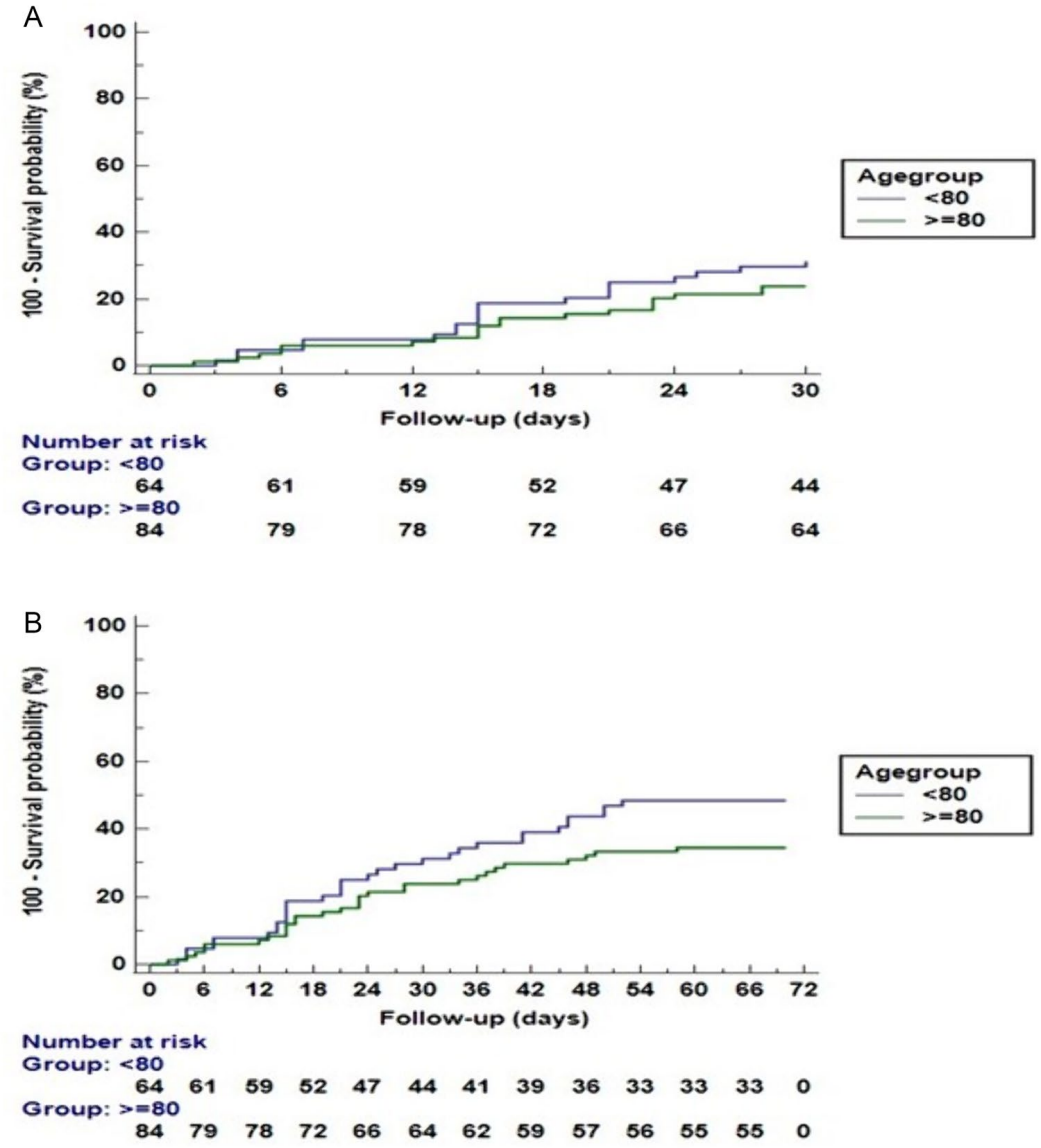
Kaplan–Mayer curve of cumulative readmission/mortality frequency in 30 (**A**) and 60 (**B**) days after discharge. **A** Readmission/mortality within 30 days. Log rank: *p* = 0.310. **B** Readmission/mortality within 60 days. Log rank: *p* = 0.100

*Clinical outcomes:* major clinical outcomes during hospitalization and in 30 days after discharge are summarized in Table 3. The duration of hospital stay was significantly shorter in the group of very elderly compared to the group of elderly (10.5 vs. 19.5 days *p* < 0.001). ICU admission frequency was significantly higher in the group of elderly compared to the group of very elderly (10.7% vs. 1.8% *p* = 0.01). No significant difference was found in the frequency of in-hospital mortality (Table 3). None of the patients in the cohort was defined as suffering from toxic megacolon, and no colectomy case was documented. Renal replacement therapy was initiated in two cases from the group of elderly and none in the group of very elderly. Amongst the patients that were discharged from hospital, there was no significant difference in mortality, the composite of readmission/mortality and in readmission due to CDI, Table 3. A Kaplan–Meir curve of 30- and 60-days post-discharge composite readmission/mortality is depicted in Fig. 1 panel A and panel B, respectively.

**Table 3:** In-hospital and 30 days post-discharge major clinical outcomes

	< 80 years (elderly)	≥ 80 years (very elderly)	
In-hospital outcome	*N* = 84	*N* = 112	*p* value
LOS (days, median IQR)	19.5 (9; 35.8)	10.5 (7; 16.8)	< 0.001
ICU admission *N* (%)	9 (10.7%)	2 (1.8%)	0.01
Mortality *N* (%)	20 (24%)	28 (25%)	0.85

30 days post-discharge outcome	*N* = 64	*N* = 84	
Mortality *N* (%)	3 (4.7%)	8 (9.5%)	0.26
Readmission/mortality *N* (%)	20 (31.3%)	20 (23.8%)	0.31
CDI-related readmission *N* (%)	4 (6.2%)	6 (7.1%)	0.99

*LOS* length of stays, *ICU* intensive care unit, *CDI* clostridioides difficile infection

The association between baseline patient characteristics and in-hospital mortality is presented in Table 4. In univariate analysis, male gender, leukocyte count, neutrophil percentage, creatinine, albumin, and CRP were found to be significantly associated with in-hospital mortality. On a multivariate analysis, creatinine, albumin, and neutrophil percentage were found to be associated with increased in-hospital mortality. Age was not found to be associated with increased in-hospital mortality in univariate and multivariate analyses.

**Table 4:** In-hospital mortality, univariate, and multivariate analyses

In-hospital mortality			
Univariate analysis	No	Yes	*p* value
	*N* = 148	*N* = 48	
Age > 80 years *N* (%)	84 (56.8)	28 (58.3)	0.848
Male gender *N* (%)	47 (31.8)	23 (47.9)	0.042
Malignancy *N* (%)	51 (34.5)	15 (31.3)	0.683
Diabetes *N* (%)	58 (39.2)	23 (47.9)	0.286
Dementia *N* (%)	39 (26.4)	15 (31.3)	0.509
IHD *N* (%)	71 (48.0)	23 (47.9)	0.995
HF *N* (%)	45 (30.4)	20 (41.7)	0.15
Hypertension *N* (%)	121 (81.8)	42 (87.5)	0.335
CVA *N* (%)	40 (27.0)	14 (29.2)	0.773
COPD *N* (%)	18 (12.2)	9 (18.8)	0.25
CRF *N* (%)	68 (45.9)	19 (39.6)	0.441
Disability *N* (%)	73 (49.3)	25 (52.1)	0.74
Leucocytes median (IQR)	11 (8; 15.3)	13.5 (10; 19.5)	0.019
Neutrophils% median (IQR)	81 (72; 87)	87.5 (80; 90)	< 0.001
Creatinine median (IQR)	1.1 (0.8; 1.9)	1.7 (0.95; 2.5)	0.007
Albumin median (IQR)	2.9 (2.5; 3.3)	2.5 (2; 2.9)	< 0.001
CRP median (IQR)	8.1 (3.4; 15.5)	13.3 (8.6; 21)	0.004
Charlson score median (IQR)	7 (5; 9) *N* = 134	7 (5; 9) *N* = 43	0.945

Multivariate analysis	OR 95% CI	*p* value
Age group (≥ 80 vs. < 80)	1.26 (0.59–2.68)	0.54
Gender	0.62 (0.3–1.29)	0.2
Leukocytes	0.99 (0.93–1.04)	0.6
Neutrophils %	1.06 (1.01–1.12)	0.01
Creatinine	11.16 (0.94–1.05)	0.014
Albumin	0.31 (0.16–0.61)	< 0.001
CRP	1.01 (0.97–1.05)	0.54

*IHD* ischemic heart disease, *HF* heart failure, *CRF* chronic renal failure, *CVA* cerebrovascular accident, *COPD* chronic obstructive pulmonary disease, *CRP* *c*-reactive protein, *SE* standard error

The association between longer hospital stays (≥ 10 days) and baseline patients’ characteristics in the group that survived the initial hospitalization is presented in Table 5. In univariate analysis, age ≥ 80, a male gender, comorbidity with chronic obstructive pulmonary disease (COPD), and lower albumin levels were associated with longer hospital stays in univariate analysis. In a multivariate analysis, COPD, and lower albumin levels were associated with longer hospital stays.

**Table 5:** Baseline patient characteristics and length of hospital stays, univariate and multivariate analyses

	Length of hospital stays		
Univariate analysis	10 ≤ days	> 10 days	*p* value
	*N* = 65	*N* = 83	
Age > 80 years *N* (%)	44 (67.7)	40 (48.2)	0.017
Male gender *N* (%)	16 (24.6)	31 (37.3)	0.042
Malignancy *N* (%)	17 (26.2)	34 (41.0)	0.06
Diabetes *N* (%)	25 (38.5)	33 (39.8)	0.873
Dementia *N* (%)	22 (33.8)	17 (20.5)	0.067
IHD *N* (%)	32 (49.2)	39 (47.0)	0.786
HF *N* (%)	1 (27.7)	27 (32.5)	0.525
Hypertension *N* (%)	53 (81.5)	68 (81.9)	0.951
CVA *N* (%)	17 (26.2)	23 (27.7)	0.832
COPD *N* (%)	3 (4.6)	15 (18.1)	0.013
CRF *N* (%)	30 (46.2)	30 (45.8)	0.964
Disability *N* (%)	34 (52.3)	39 (47.0)	0.521
Leucocytes median (IQR)	11 (8.4; 15.8)	10.6 (7.6; 15)	0.624
Neutrophils% median (IQR)	81 (71; 86)	81 (72; 87)	0.835
Creatinine median (IQR)	1.2 (0.8; 1.9)	1.1 (0.74; 1.9)	0.387
Albumin median (IQR)	3.1 (2.6; 3.5)	2.9 (2.3; 3.2)	0.009
CRP median (IQR)	6.5 (2.7; 15.3)	8.7 (5; 16)	0.182
Charlson score median (IQR)	8 (5.8; 9) *N* = 58	7 (5; 9) *N* = 76	0.788

Multivariate analysis	OR 95% CI	*p* value
Age group (≥ 80 vs. < 80)	0.5 (0.23–1.06)	0.069
Gender (female vs. male)	0.56 (0.25–1.27)	0.162
Malignancy	1.99 (0.91–4.3)	0.084
COPD	5.8 (1.5–23.2)	< 0.001
Dementia	0.68 (0.29–1.63)	0.39
Albumin	0.39 (0.21–0.72)	0.002

*IHD* ischemic heart disease, *HF* heart failure, *CRF* chronic renal failure, *CVA* cerebrovascular accident, *COPD* chronic obstructive pulmonary disease, *CRP* *c*-reactive protein, *SE* standard error

## Discussion

In the current study, we found shorter hospital stays and less frequent ICU admissions in the very elderly compared to elderly but no difference in in-hospital and post-discharge mortality. The patients in our cohort are predominantly of advanced age with a mean age of 78.5 years. The context given is that our hospital is in the city of Haifa, which is known for having a population that skews towards older individuals compared to the rest of Israel. According to the Israel National Insurance Institute, 24.2% of Haifa population are senior citizens, compared to 14.9% in the general Israeli population. Moreover, within the city of Haifa, the Carmel area (where our hospital is located) is known to be a home to a large population of very elderly individuals. Thus, although our data may reflect a “real-life” situation, it may not represent a “real-world” cohort. Intuitively, comorbidities tend to become more common as people age. This is largely because various health conditions accumulate over time due to factors like natural aging processes and cumulative exposure to risk factors, such as poor diet, lack of exercise, smoking, or environmental toxins. These factors can contribute to the development of chronic conditions like diabetes, hypertension, or heart diseases. In our cohort, ischemic heart disease, dementia, and disability were more prevalent in the very elderly whereas diabetes and chronic renal failure seemed to be more prevalent in the elderly. The Charlson morbidity index score was understandably higher in the very elderly. These findings may suggest “natural selection bias” between the two groups. Those who reach an advanced age often possess certain characteristics that have contributed to their longevity. These characteristics may include genetic factors that make them more resilient to various diseases and health conditions, healthier lifestyles, and better access to healthcare [22]. Thus, the possible presence of these factors in the very elderly demonstrates that chronologic age is not the only factor that influences outcomes in acute and chronic illnesses including CDI.

Although there was no difference in the occurrence of severe-complicated CDI between the elderly and very elderly, the “younger” patients were admitted more frequently to ICU. The decision to admit a patient to the ICU is a complex and critical one, particularly in healthcare systems with limited resources. ICU care is associated with high costs, and potential complications. Several factors influence this decision, with a primary consideration being the evaluation of the patient’s overall chances of survival and the potential for a meaningful recovery, in addition to ICU bed availability, patient and family preferences, and ethical considerations [23]. There are no age threshold criteria for ICU admission in our institution. However, given that the major clinical outcomes were not different between the groups, this may raise a concern about age-based bias in ICU admission.

The in-hospital and post-discharge survival were not decreased in the very elderly patients compared to the elderly. Given fewer ICU admissions in this group, this may suggest that care in the internal medicine department may at least be non-inferior to ICU in very old patients. In recent decades, there has been a noticeable trend towards older patients being admitted to internal medicine departments [24]. Several factors contribute to this demographic shift: extended life expectancy, increased prevalence of chronic conditions, advances in medical care and technology that enables many individuals to live longer with chronic diseases that can seek care in internal medicine departments. Internists are expected to manage a wide spectrum of health issues, including chronic diseases, acute illnesses, complex medical conditions, and polypharmacy. Hence, the department of internal medicine became well-suited for providing comprehensive care for elderly patients.

In-hospital mortality in our cohort is consistent with that reported in the literature, varying between 8 and 37.2% [25]. Several factors contribute to this variability, including the severity of infection, patient characteristics, diagnostic and therapeutic approaches, and differences in data collection and reporting.

The baseline laboratory markers that were associated with increased in-hospital mortality in our study included higher creatinine, lower albumin, and neutrophilia-findings that are consistent with reported literature [26, 27].

The frequency of toxic megacolon necessitating colectomy in patients with CDI is not clearly reported in the literature. In patients with fulminant CDI, early colectomy was associated with improved survival [28, 29]. None of our patients underwent colectomy. We also found no documentation that colectomy was considered in any of our cases. These findings may suggest that we need to increase our awareness of this potentially life-saving intervention.

In multivariate analysis, we found that COPD comorbidity and lower albumin levels were associated with longer hospital stays whereas age was not. Further research and a more detailed analysis of these factors may be needed to better understand their impact on hospitalization. Interestingly, we found that there was no association between other comorbidities and the Charlson score and longer hospital stays.

This is a retrospective study with obvious limitations. A large portion (~ 35%) of the screened records had to be excluded due to lack of proper documentation or missing CDI toxin test results. This finding highlights the potential drawbacks associated with automated electronic data extraction. Medical data on a single subject accumulate overtime and often lack routine update. Moreover, the quality of medical documentation in a real-life setting is sometimes not optimal. In this study, we believe that case-by-case thorough verification of data was needed to ensure data quality. Another limitation in our study is that data collection was started in 2019 and the last patient included in our cohort in 2018. This may reflect the challenges faced by clinicians in an acute setting, particularly in the internal medicine department, regarding time and resources for conducting, improving, and disseminating quality clinical research. In a fast-paced and high-demand environment, clinicians often encounter several obstacles when it comes to engaging in research activities and to producing high quality publication in a timely manner [30]. Noteworthy, geriatric patients admitted to the hospital are prone to numerous other complications besides CDI, such as other nosocomial infections, deep vein thrombosis, falls, delirium, etc. These are potential confounders that were not addressed in our study.

In conclusion, although admitted less frequently to ICU, in our cohort the in-hospital survival of the very elderly was not adversely affected compared to the elderly patients with CDI. This observation, along with decreased duration of hospital stay and the extended life expectancy observed in the general population, may suggest that chronological age should not be the main determinant of the overall prognosis in very elderly patients with CDI. Larger and prospective observations may be needed to further characterize CDI morbidity in the very elderly.

## Data Availability

Not applicable.
